# pH responsive alginate polymeric rafts for controlled drug release by using box behnken response surface design

**DOI:** 10.1080/15685551.2016.1231046

**Published:** 2016-09-11

**Authors:** Ghulam Abbas, Muhammad Hanif, Mahtab Ahmad Khan

**Affiliations:** ^a^ Faculty of Pharmacy, Bahauddin Zakariya University, Multan, Pakistan; ^b^ Faculty of Pharmaceutical Sciences, Government College University Faisalabad, Pakistan

**Keywords:** Sodium alginate, box-behnken design, raft resilience, *in vitro* drug release, FTIR, DSC, XRD

## Abstract

Aim of the present work was to develop alginate raft forming tablets for controlled release pantoprazole sodium sesquihydrate (PSS). Box behnken design was used to optimize 15 formulations with three independent and three dependent variables. Physical tests of all formulations were within pharmacopoeial limits. Raft was characterized by their strength, thickness, resilience, acid neutralizing capacity, floating lag time and total floating time. Raft strength, thickness and resilience of optimized formulation AR9 were 7.43 ± 0.019 g, 5.8 ± 0.245 cm and greater than 480 min, respectively. Buffering and neutralizing capacity were 11.2 ± 1.01 and 6.5 ± 0.56 meq, respectively. Dissolution studies were performed by using simulated gastric fluid pH 1.2 and cumulative percentage release of optimized formulation AR9 was found 98%. First order release kinetics were followed and non-fickian diffusion was observed as value of n was greater than 0.45 in korsmeyer-peppas model. PSS, polymers, tablets and rafts were further characterized by Fourier transform infrared spectroscopy (FTIR), X-ray diffractometry (XRD) and differential scanning calorimetry (DSC). FTIR spectra of PSS, polymers and raft of optimized formulation AR9 showed peaks at 3223.09, 1688.17, 1586.67, 1302.64 and 1027.74 cm^−1^ due to –OH stretching, ester carbonyl group (C=O) stretching, existence of water and carboxylic group in raft, C–N stretching and –OH bending vibration showed no interaction between them. XRD showed diffraction lines indicates crystalline nature of PSS. DSC thermogram showed endothermic peaks at 250 °C for PSS. The developed raft was suitable for controlled release delivery of PSS.

## 1. Introduction

The advancement of new materials built on polysaccharides is due to their benefits as low cost, freely available, biodegradable, non-toxic and sustainability. Biopolymers like sodium alginate, pectin and numerous others have been used in the field of GRDD. Sodium alginate, the sodium salt of alginic acid, is a biodegradable non-toxic naturally occurring macromolecule hydrate and swells in water but in acidic environment it produces gel after protonation.[1] Alginate consists of linear copolymers of 1, 4-glycosidically linked β-D-mannuronic acid and α-L-guluronic acid. Sodium alginate, a pH sensitive polymer stable at acidic pH but unstable in alkaline medium because at higher pH a rapid dissolution occur that limits its application and can be crosslinked by physical and chemical mechanisms. Mono and divalent cations (sodium and calcium) can be used for crosslinking of sodium alginate to form three dimensional gel network.[2,3] Hydroxypropyl methyl cellulose K100M (HPMC K100M) a hydrophilic polymer sustained the release of drug by increasing the viscosity of gel layer. HPMC K100M releases the drug from gel barrier by diffusion process.[4]

Previously reported rafts such as alginate rafts of gaviscon liquid do not neutralize the gastric acid but inside the raft high pH was maintained for an extended period of time. Hampson et al. reported the alginate rafts and their various parameters used for the characterization such as raft resilience or resistance and buoyancy.[5] The addition of antacids such as aluminum hydroxide have negative effect on structure and strength of raft but calcium carbonate have positive effect on raft thickness and strength. Hampson et al. reported the effect of antacid on raft structure and strength.[6] In 2014 Jang et al. develop the risedronate sodium raft for reduction of esophageal irritation by using sodium alginate as raft forming polymer.[7] Raft of curcumin-eudragit by using sodium alginate as gelling polymer and calcium carbonate for generating CO_2_ and Ca^2+^ reported by Kerdsakundee et al. [8]

The objective of this research work is to develop and characterize alginate rafts for the treatment of peptic ulcer along with reflux disorders. *In vitro* modified balance method will be developed for measurement of strength of raft. Modified paddle mixer apparatus is developed for the determination of raft resilience. Acid neutralizing and buffering capacity of sodium bicarbonate and citric acid are measured by modified USP type II dissolution apparatus and effect of calcium on strength of raft is determined. The developed formulations are further characterized by determining the floating lag time (FLT) and total floating time (TFT) of raft. *In vitro* dissolution studies are performed to check the release pattern of pantoprazole sodium sesquihydrate (PSS). Fourier transform infrared spectroscopy (FTIR) studies are performed to check the interactions between drug, polymers and other excipients. X-ray diffractometry (XRD) is used to check the crystalline or amorphous nature of the drug and polymers and differential scanning calorimetry (DSC) is used to check the thermal behavior of drug and polymers.

## 2. Experimental

### 2.1. Materials

PSS was obtained as a gift sample from Shrooq Pharmaceuticals Pvt. Ltd. Lahore, Pakistan. Sodium alginate and HPMC K100M were of analytical grade and purchased from Sigma–Aldrich Chemie Gmbh Germany. Sodium bicarbonate, citric acid, and calcium carbonate were obtained from KGaA Darmstadt, Germany. Pepsin was obtained from Scharlau Barcelona, Spain. Double distilled water was used in whole study and other chemicals/reagents used was of analytical grade.

### 2.2. Methods

#### 2.2.1. Box behnken design

Box behnken response surface designs are used to require three levels, coded as −1, 0, and +1. Box behnken design (BBD) was used [9] for optimization of tablets having three independent variables and three dependent variables using design expert (version 7.1 state-ease Inc., Minneapolis, MN). Independent variables were percentages of sodium alginate (*X*_1_), HPMC K100M (*X*_2_) and sodium bicarbonate (*X*_3_) while the dependent variables were % drug release of PSS at 2 h (*Y*_2_), 4 h (*Y*_4_) and at 8 h (*Y*_8_) as shown in Table 1. The nonlinear quadratic model by this design is given as [10];

(1) $$Yi=b_{0}+b_{1}X_{1}+b_{2}X_{2}+b_{3}X_{3}+b_{12}X_{1}X_{2}+b_{13}X_{1}X_{3}+b_{23}X_{2}X_{3}+b_{11}X_{1}^{2}+b_{22}X_{2}^{2}+b_{33}X_{3}^{2}$$

**Table 1.** Independent and dependent variables and constrains in box-behnken design.

Variables	Level			Constrains
	−1	0	+1	
Independent				
*X*_1_ Sodium alginate (%)	10	25	40	In the range
*X*_2_ HPMC K100M (%)	6	10	14	In the range
*X*_3_ Sodium bicarbonate (%)	20	30	40	In the range
Dependent				
*Y*_2%_ drug release at 2 h (%)				20–40
*Y*_4%_ drug release at 4 h (%)				40–60
*Y*_8%_ drug release at 8 h (%)				80–100

where *Yi* is the measured response of the dependent variables, *b*_0_ is the intercept, *b*_1_–*b*_33_ are the regression coefficients computed from the observed experimental values of *Y*. *X*_1_, *X*_2_ and *X*_3_ are the coded value of the independent variables. *X*_*a*_ *X*_*b*_ (*a*, *b* = 1, 2, 3) and $X_{i}^{2}$ (*i* = 1, 2, 3) represent the interaction and quadratic terms, respectively.

#### 2.2.2. Preparation of tablets

Tablets were prepared by mixing PSS, sodium alginate, HPMC K100M (for sustained release effect), sodium bicarbonate, citric acid and calcium carbonate by using sigma mixer and passed through 20-mesh screen. Composition of 15 formulations are given in Table 2. Powder blend passed from the micromeritic limits were mixed thoroughly for 5 min by using sigma mixer. The mixture was granulated using 2% (w/w) HPMC E5 in a 90% ethanol solution. 2% (w/w) HPMC E5 in 90% ethanol used as granulating agent. The prepared granules were dried at 40 °C for 2 h, passed through 18-mesh screen.[4,11] Granules were compressed by using minipress MII (pharma test Hainburg, Germany). Physical tests of tablets such as weight variation, hardness, thickness, diameter and friability were performed.

**Table 2.** Composition of alginate raft forming tablets.

Formulation code	PSS (mg)	Sodium alginate (mg)	HPMC K100M (mg)	Sodium bicarbonate (mg)	Citric acid (mg)	Calcium carbonate (mg)	Total weight (mg)
AR1	40	40	56	120	60	84	400
AR2	40	100	40	120	60	40	400
AR3	40	160	40	100	50	10	400
AR4	40	40	40	80	40	160	400
AR5	40	100	40	120	60	40	400
AR6	40	120	56	100	50	34	400
AR7	40	100	24	80	40	116	400
AR8	40	140	24	120	60	16	400
AR9	40	160	40	80	40	40	400
AR10	40	100	24	140	70	26	400
AR11	40	40	24	120	60	116	400
AR12	40	100	40	120	60	40	400
AR13	40	80	56	140	70	14	400
AR14	40	40	40	160	80	40	400
AR15	40	100	56	80	40	84	400

#### 2.2.3. Effect of pH on raft formation

Prepared tablets were added into 900 ml of simulated gastric fluid (SGF) having pH 1.2, 5.8, 1.0 N HCl pH 1.2 and 0.1 N HCl pH 5.7 and effect of pH on raft formation was observed.[12]

#### 2.2.4. Disintegration time of tablet in water

Disintegration time was measured by placing one tablet in 120 ml of distilled water at room temperature and evaluation of gas around the tablet or its fragments were observed. Tablet was fragmented if the evolution of gas around the tablet or its fragments stopped, being either dissolved or dispersed in water so that no agglomerate remains. The same process was repeated on four additional tablets.[7,13]

#### 2.2.5. Raft strength

Prepared tablet was transferred to 150 ml of SGF pH 1.2 at 37 °C. SGF was prepared with 2.0 g of sodium chloride, 3.2 g of purified pepsin and 7 ml of HCl in 1000 ml of distilled water. Raft was allowed to form around L-shaped wire probe (diameter: 1.2 mm) held straight in the beaker for 30 min. Raft strength was measured by using the modified balance method.[13]

#### 2.2.6. Volume, weight and thickness of raft

Tablet was transferred to 150 ml of SGF pH 1.2 maintained at 37 °C and wait of 30 min until the raft was formed. Beaker used for raft formation was pre-weighed (*W*_1_). Top of each raft was observed from outer surface of beaker. The whole weight of beaker and filling was obtained after raft formation (*W*_2_). Raft was removed from the beaker by pouring off the subnatant liquor and weighed (*W*_3_). Remaining liquid was removed from the beaker and it was refilled with water to the noticeable position and weighed (*W*_4_). The volume of each raft was measured in ml and weight was measured in grams.[5] Thickness of raft was measured by placing tablet in 150 ml of SGF. Raft was allowed to form for 10 min and thickness of the raft was measured at three places around the cylinder by using digital vernier caliper (Shandong, China) and expressed as mean value.[7]

#### 2.2.7. Raft resilience

Place one tablet in 150 ml of SGF pH 1.2 at 37 °C in 250 ml glass jar and wait for 30 min until the raft was completely developed. Glass jar was capped and positioned in modified tumble mixer, set to revolve at 20 rpm, to simulate gastric agitation. Raft was assessed visually for such time that a raft could no longer be noticed. A raft was distinct or dispersed into two or more hovering gels at least 15 mm in diameter.[6]

#### 2.2.8. FLT and TFT

USP dissolution apparatus II (pharma test Hainburg, Germany) was used for the determination of FLT and TFT. Add one tablet in 900 ml SGF pH 1.2 maintained at 37 ± 0.5 °C and set at 50 rpm. The time required for raft to rise to the surface and float was determined as FLT. TFT is the total time for which the raft floats in dissolution medium including FLT.[8]

#### 2.2.9. Acid neutralization capacity

The acid neutralization ability of raft forming tablet was estimated using an *in vitro* method. The dissolution apparatus II (paddle method) was operated with a paddle speed of 125 rpm and with 250 ml of 0.02 M HCl solution at 37 °C. Tablet formulation dissolved solution 120 ml was added into the medium and the pH of the medium was checked continuously, after 20 min the burette started with continuous titration of 0.1 M HCl solution at a continual speed of 2.0 ml/min until the acidity of medium reached pH 2.5.Neutralizing and total buffering capacity from pH 2.5–4.5 was calculated by following equations.

(2) $$\text{Neutralization capacity}=\left[\left(V_{\text{HCl}}\times T_{\text{HCl}}\right)+\left(V_{tr-2}\times T_{tr}\right)\right]\times \frac{W1}{W2}$$

(3) $$\text{Buffering capacity}=\left(V_{tr-1}\times T_{tr}\right)\times \frac{W1}{W2}$$

where *V*_HCl_ is the volume of HCl in the vessel, *T*_HCl_ the titer of HCl in the vessel, *V*_*tr*−2_ the added volume of HCl from the burette until pH 2.5, *T*_tr_ the titer of HCl in the burette, *W*_1_ the weight of intact formulation and *W*_2_ the weight of tested quantity of formulation. *V*_*tr*−1_ is the added volume of HCl from the burette between pH 2.5 and 4.5, *T*_*tr*_ the titer of HCl in the burette.[7]

#### 2.2.10. *In vitro* drug release studies

The *in vitro* drug release study was carried in 900 ml SGF of pH 1.2 at 37 ± 0.5 °C from 0 to 8 h by using USP dissolution apparatus II at 50 rpm. 5 ml aliquot was pipette out at regular interval and replaced with fresh medium of same volume. The aliquot was filtered by 0.45 μm filter and concentration of drug was determined by UV spectrophotometer (PerkinElmer Inc. New York, USA) at 290 nm.[14]

#### 2.2.11. Drug release kinetics

The mechanisms of controlled release alginate raft forming formulations were determined by different *in vitro* kinetics models such as zero order (Equation 4), first order (Equation 5), higuchi (Equation 6) and Korsmeyer-peppas model (Equation 7).[15]

(4) $$F=K_{0}t$$

(5) $$\ln\left(1-F\right)=-K_{1}t$$

(6) $$F=K_{2}t^{1/2}$$

(7) $$M_{t/M_{\infty }}=K_{3}t^{n}$$

where *F* is fraction of drug release in time *t*, *K*_0_ is rate constant for zero order release equation, *K*_1_ is first order release constant, *K*_2_ is higuchi constant, *M*_*t*_ is amount of drug release at time *t*, *M*_∞_ is amount of drug release at infinity and *n* is diffusion constant.

#### 2.2.12. Fourier transform infrared spectroscopy

FTIR of PSS, sodium alginate, HPMC K100M and raft of optimized formulation AR9 were obtained by FTIR spectrophotometer (Bruker Alpha, Germany) and compared. The spectra was recorded at wavelength range of 800–3500 cm^−1^.

#### 2.2.13. X-ray diffractometry

Crystalline or amorphous nature of drug, polymers, prepared tablets and rafts were evaluated from their diffractograms. Diffractograms of PSS, sodium alginate, HPMC K100M, tablet of optimized formulation AR9 and raft of AR9 optimized formulation were obtained using an XRD diffractometer D/max-2500pc, Rigaku Co, Japan. Tube voltage was 40 kV, current was mA, and scanning rate was 5^0^ over a range of 80–800 diffraction angle.

#### 2.2.14. Differential scanning calorimetry

DSC was used to analyze the thermal characteristics of the powdered sample of drug and polymers, physical mixture, prepared tablets and raft. DSC thermograms of PSS, sodium alginate, HPMC K100M, tablet of optimized formulation and raft of AR9 optimized formulation were obtained by using differential scanning calorimeter DSC-60 Shimadzu, Germany. 5.5 mg sample was placed in aluminum pans, sealed and analyzed under a stream of nitrogen gas of 100 ml/min and heated from 50 to 350 °C.

## 3. Results and discussion

**Figure 1.**
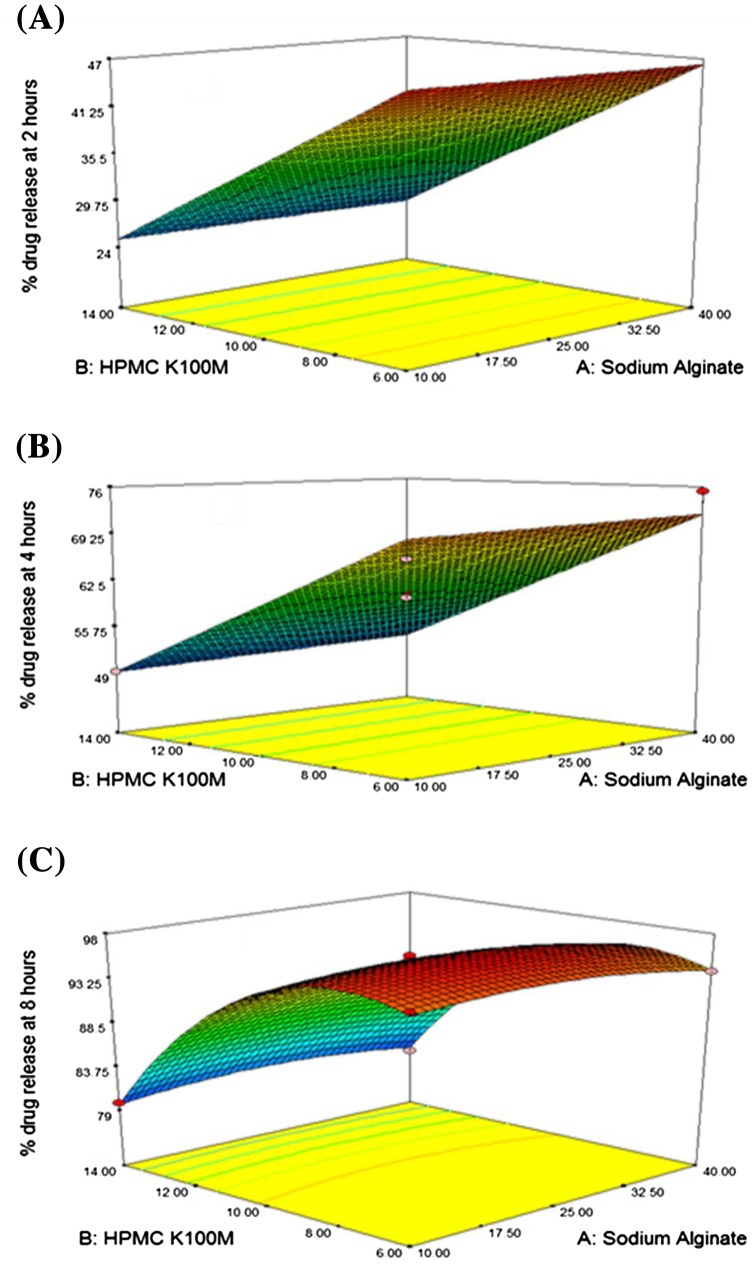
3D response surface graph showing effects of sodium alginate (*X*_1_), HPMC K100M (*X*_2_) and sodium bicarbonate (*X*_3_) on (A) % drug release at 2 h (*Y*_2_), (B) % drug release at 4 h (*Y*_4_) and (C) % drug release at 8 h (*Y*_8_).

**Figure 2.**
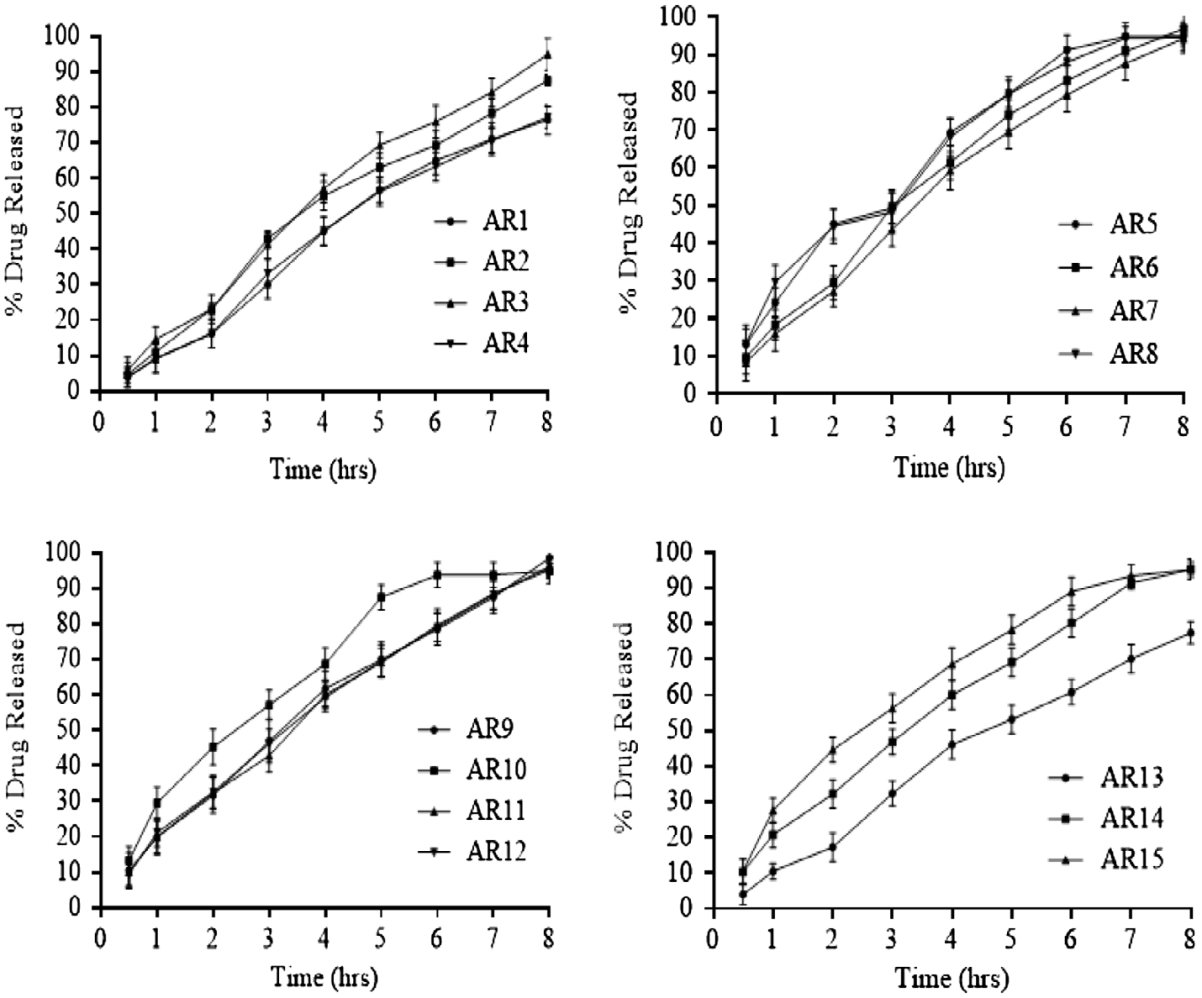
Drug release profile of pantoprazole sodium sesquihydrate from alginate rafts (*n* = 6).

**Figure 3.**
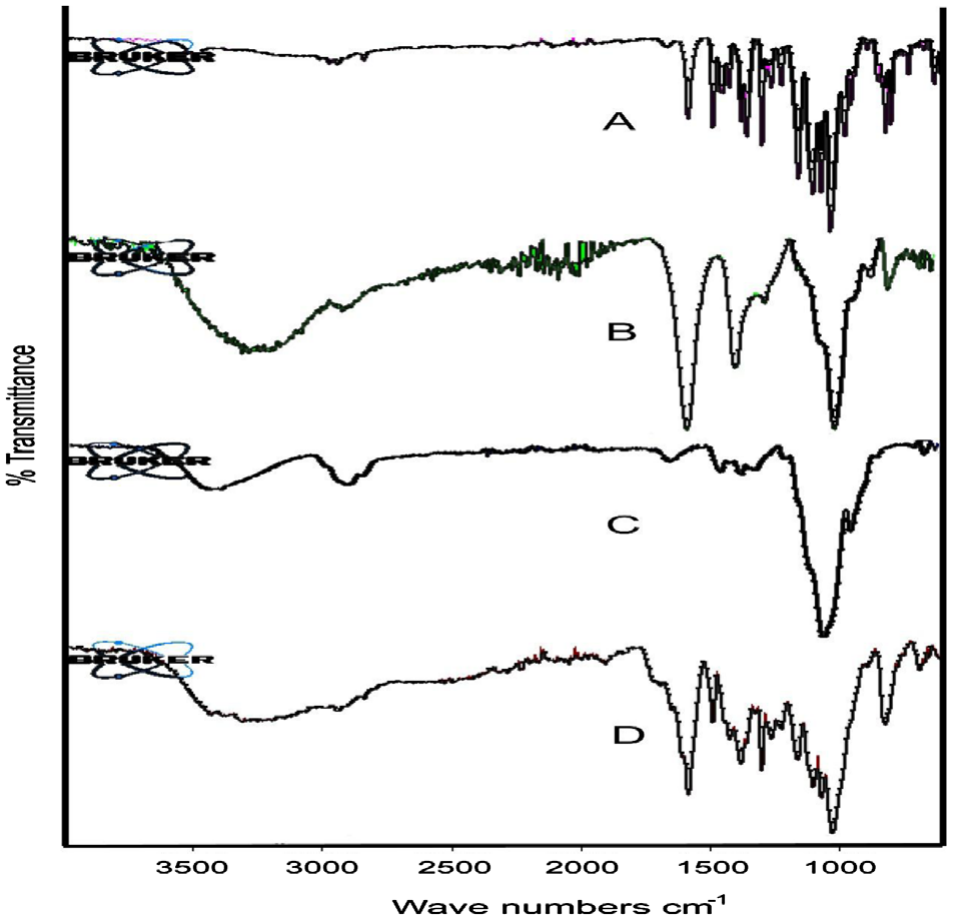
FTIR spectra of (A) PSS, (B) sodium alginate, (C) HPMC K100M and (D) alginate raft of optimized formulation AR9.

**Figure 4.**
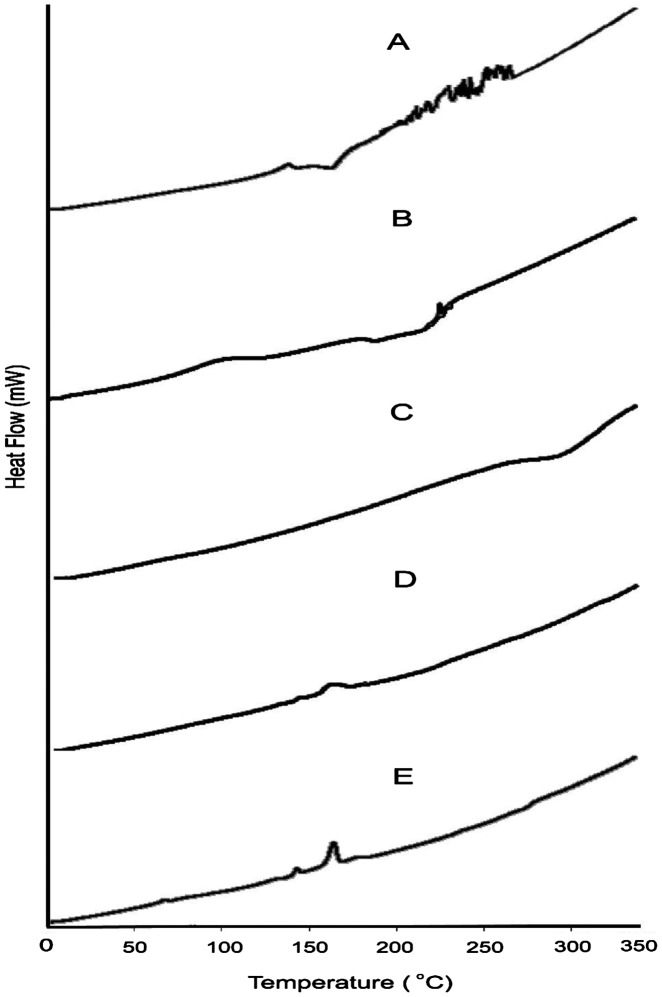
DSC thermograms of (A) PSS, (B) sodium alginate, (C) HPMC K100M and (D) tablet of optimized formulation AR9 and (E) alginate raft of optimized formulation AR9.

**Figure 5.**
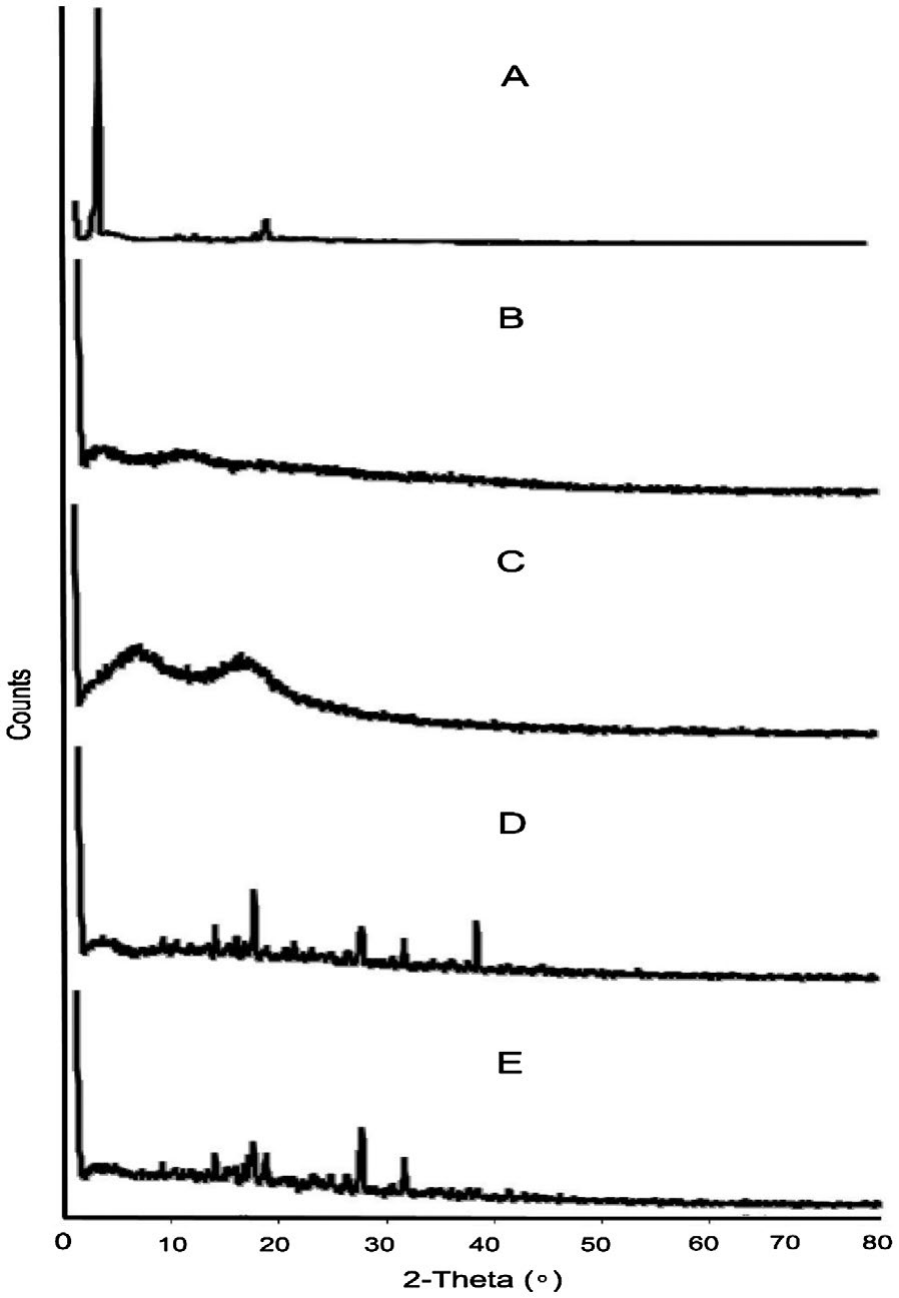
XRD of (A) PSS, (B) sodium alginate, (C) HPMC K100M and (D) tablet of optimized formulation AR9 and (E) alginate raft of optimized formulation AR9.

Interaction between independent and dependent variables (Table 3) were studied and three dimensional graphs were developed as shown in Figure 1. Disintegration time of tablets and strength, weight, volume and thickness of rafts were within pharmacopoeial limits are mentioned in Table 4. Effect of different pH medium on raft formation was studied successfully. Buffering capacity, neutralizing capacity, resilience, FLT and TFT of rafts of all formulations were successfully determined and are shown in Table 5. The release pattern of PSS form pectin rafts were determined and are shown in Figure 2. FTIR spectra of PSS, sodium alginate, HPMC K100M and raft of optimized formulation AR9 showed compatibility of PSS with polymers and are shown in Figure 3. DSC thermograms and X-ray diffractograms of PSS, sodium alginate, HPMC K100M, tablet of optimized formulation AR9, and raft of optimized formulation AR9 showed compatibility of drugs with polymers and are shown in Figures 4 and 5, respectively.

**Table 3.** Observed responses for alginate rafts forming tablets (*n* = 6).

Formulation code	Independent variables			Dependent variables		
	*X*_1_ (%)	*X*_2_ (%)	*X*_3_ (%)	*Y*_2_ (%)	*Y*_4_ (%)	*Y*_8_ (%)
AR1	10	14	30	24.54 ± 0.011	49.11 ± 0.023	79.78 ± 0.091
AR2	25	10	30	35.43 ± 0.023	60.98 ± 0.982	95.67 ± 0.095
AR3	40	10	25	31.12 ± 0.123	53.89 ± 0.312	90.12 ± 0.011
AR4	10	10	20	41.23 ± 0.093	64.65 ± 0.256	93.11 ± 0.034
AR5	25	10	30	36.87 ± 0.034	59.92 ± 0.095	95.23 ± 0.711
AR6	30	14	25	30.99 ± 0.081	57.78 ± 0.367	79.12 ± 0.098
AR7	25	6	20	47.76 ± 0.456	72.21 ± 0.087	92.68 ± 0.458
AR8	35	6	30	47.34 ± 0.125	75.29 ± 0.087	94.01 ± 0.059
AR9	40	10	20	36.50 ± 0.087	58.89 ± 0.054	98.32 ± 0.911
AR10	25	6	35	44.42 ± 0.056	71.12 ± 0.178	95.32 ± 0.081
AR11	10	6	30	45.67 ± 0.049	68.34 ± 0.034	95.67 ± 0.487
AR12	25	10	30	34.44 ± 0.086	64.55 ± 0.031	94.42 ± 0.043
AR13	20	14	35	25.65 ± 0.056	46.65 ± 0.012	79.35 ± 0.045
AR14	10	10	40	33.99 ± 0.014	58.87 ± 0.054	94.45 ± 0.123
AR15	25	14	20	21.53 ± 0.987	46.55 ± 0.654	81.98 ± 0.014

**Table 4.** Disintegration time of tablets and strength, weight, volume and thickness of raft (*n* = 6).

Formulation code	Disintegration time (s)	Raft strength (g)	Raft weight (g)	Raft volume (ml)	Raft thickness (cm)
AR1	54	3.19 ± 0.067	1.39 ± 0.012	5.5 ± 0.15	3.5 ± 0.045
AR2	63	5.29 ± 0.039	1.84 ± 0.010	7.2 ± 0.05	4.5 ± 0.216
AR3	57	7.31 ± 0.012	2.10 ± 0.029	8.9 ± 0.05	5.5 ± 0.136
AR4	55	3.10 ± 0.097	1.32 ± 0.011	5.7 ± 0.25	3.9 ± 0.048
AR5	67	5.20 ± 0.013	1.85 ± 0.010	7.5 ± 0.15	4.7 ± 0.193
AR6	65	7.30 ± 0.067	2.20 ± 0.013	8.4 ± 0.35	5.6 ± 0.085
AR7	64	5.59 ± 0.019	1.79 ± 0.011	7.3 ± 0.25	4.7 ± 0.212
AR8	63	7.32 ± 0.047	2.00 ± 0.015	8.7 ± 0.15	5.5 ± 0.110
AR9	64	7.11 ± 0.010	2.19 ± 0.010	8.8 ± 0.05	5.8 ± 0.245
AR10	57	5.10 ± 0.062	1.80 ± 0.034	7.0 ± 0.45	4.3 ± 0.021
AR11	53	3.11 ± 0.069	1.23 ± 0.011	5.9 ± 0.85	3.8 ± 0.125
AR12	57	5.78 ± 0.067	1.79 ± 0.032	7.9 ± 0.14	4.9 ± 0.211
AR13	54	5.61 ± 0.076	1.90 ± 0.021	7.8 ± 0.25	4.7 ± 0.745
AR14	53	3.15 ± 0.013	1.11 ± 0.012	5.5 ± 0.15	3.4 ± 0.236
AR15	59	5.36 ± 0.063	1.92 ± 0.056	7.6 ± 0.15	4.6 ± 0.045

**Table 5.** Buffering capacity, neutralizing capacity, resilience, FLT and TFT of raft forming tablets (*n* = 6).

Formulation code	pH After 4 min	pH After 20 min	Buffering capacity (meq)	Neutralizing capacity (meq)	Raft resilience (min)	FLT (s)	TFT (h)
AR1	4.1	5.4	11.5 ± 1.01	6.9 ± 0.57	>480	51	>8
AR2	4.2	5.5	11.0 ± 1.04	5.5 ± 0.49	>480	52	>8
AR3	5.5	6.7	15.7 ± 1.05	7.5 ± 0.31	>480	51	>8
AR4	3.5	4.2	10.3 ± 1.81	6.8 ± 0.55	>480	54	>8
AR5	4.6	5.6	13.9 ± 1.07	7.6 ± 0.10	>480	52	>8
AR6	4.4	5.2	11.5 ± 1.05	5.7 ± 0.49	>480	49	>8
AR7	3.2	4.6	10.6 ± 1.91	6.8 ± 0.23	>480	48	>8
AR8	4.2	5.8	12.5 ± 1.41	6.7 ± 0.26	>480	50	>8
AR9	3.8	4.9	11.2 ± 1.01	6.5 ± 0.56	>480	55	>8
AR10	5.7	6.9	14.7 ± 1.05	7.6 ± 0.12	>480	54	>8
AR11	4.1	5.0	10.90 ± 1.30	4.9 ± 0.49	>480	49	>8
AR12	4.6	5.2	13.69 ± 1.04	7.8 ± 0.16	>480	56	>8
AR13	5.0	6.3	12.10 ± 1.10	6.9 ± 0.59	>480	51	>8
AR14	5.3	6.5	10.20 ± 1.31	5.8 ± 0.49	>480	53	>8
AR15	3.4	4.3	12.70 ± 1.21	7.0 ± 0.34	>480	50	>8

The outcome of independent variables on dependent variables were studied and 3D plots were developed. Values of % drug release at 2 h were ranged from 21.53 ± 0.987 to 47.76 ± 0.456%. % drug release at 4 and 8 h were found between 46.55 ± 0.654–75.29 ± 0.087 and 79.12 ± 0.098–98.32 ± 0.911%, respectively. All responses were fitted to the quadratic models using BBD. Data of *Y*_2_, *Y*_4_ and *Y*_8_ was observed and best fitted model was quadratic and regression Equations (8–10) were generated.

(8) $$Y_{2}=35.96+0.264X_{1}-7.73X_{2}-0.83X_{3}-2.39X_{1}X_{2}-0.25X_{1}X_{3}+7.57X_{2}X_{3}+0.08X_{1}^{2}+70.05X_{2}^{2}+0.81X_{3}^{2}$$

(9) $$Y_{4}=61.19+0.29X_{1}-8.17X_{2}-0.90X_{3}-2.94X_{1}X_{2}-0.32X_{1}X_{3}+8.99X_{2}X_{3}+0.10X_{1}^{2}+81.62X_{2}^{2}+0.99X_{3}^{2}$$

(10) $$Y_{8}=95.11+0.18X_{1}-6.58X_{2}-0.75X_{3}-1.25X_{1}X_{2}-0.14X_{1}X_{3}+5.00X_{2}X_{3}+0.03X_{1}^{2}+43.85X_{2}^{2}+0.57X_{3}^{2}$$

In regression equations positive sign favors the optimization while negative sign indicates an inverse relationship between independent and dependent variables. The amount of sodium alginate (*X*_1_) HPMC K100M (*X*_2_) and sodium bicarbonate (*X*_3_) have different effects on % drug release (*Y*_2_), (*Y*_4_) and (*Y*_8_). Tested formulations showed that percentage of drug release were more when the polymers concentration were less as compared to the formulation contained high amount of polymers. When the concentration of sodium alginate and HPMC K100M were 25 and 10%, respectively the sustained release effect of drug from alginate rafts were good but below that concentrations the drug was rapidly released and above that concentrations the release of drug was slow. The interaction of *X*_1_ and *X*_2_ was insignificant and showed negative effect on *Y*_2_, *Y*_4_ and *Y*_8_ and *X*_1_ and *X*_3_ possessed negative value and have insignificant effect on *Y*_2_, *Y*_4_ and *Y*_8_. Interaction of *X*_2_ and *X*_3_ was significant and have positive effect on *Y*_2_, *Y*_4_ and *Y*_8_. Optimized formulation AR9 was selected on the basis of better release pattern of drug at 2, 4 and 8 h. Rapolu et al. studied the effect of different polymer concentrations on release profile of GRRD of metronidazole by using BBD.[9]

At pH 1.2 of SGF and 1.0NHCl tablets rapidly disintegrated and rafts were formed on the top of medium but at pH 5.7 of 0.1N HCl and 5.8 of SGF, tablets were reside at the bottom of the medium and rafts were not formed. Elliot et al. studied the effect of different pH medium on raft forming alginate-antacids combined formulations.[12]

Disintegration time of tablets of 15 formulations were ranged from 53 to 67 s. Effects of concentration of sodium alginate and sodium bicarbonate on disintegration time of tablets were observed. Formulations (AR1, AR4, AR11 and AR14) containing less amount of sodium alginate showed less disintegration time of tablets as compared to the formulations (AR3, AR6, AR8 and AR9) containing high amount of sodium alginate as shown in Table 3. Formulations (AR3, AR10, AR13 and AR14) containing higher concentration of sodium bicarbonate rapidly disintegrate as compared to the formulations (AR5, AR6 and AR7) having less amount of sodium bicarbonate. Jang et al. studied the disintegration time of risedronate sodium tablets in water containing sodium alginate as a raft forming polymer and sodium bicarbonate as gas generating substances.[7]

Raft strength was ranged from 3.10 ± 0.097 to 7.32 ± 0.047 g measured by modified balance method as shown in Table 3. Raft weight and volume was ranged from 1.11 ± 0.012 to 2.20 ± 0.013 g and 5.5 ± 0.15 to 8.9 ± 0.05 ml, respectively. Hampson et al. measured the strength, weight and volume of rafts of sodium alginate.[5] Raft thickness ranged from 3.4 ± 0.236 to 5.8 ± 0.245 cm as mentioned in Table 3. AR14 formulation showed less thickness of raft due to less amount of polymers but AR9 formulation has highest raft thickness because of maximum amount of polymers.[7] Thickness of raft was increased when the concentration of the polymers were increased. Raft resilience of all formulations were greater than 480 min as shown in Table 4.[16] Hampson et al. measured the resilience of alginate rafts and studied the effect of polymer concentration on resilience of rafts.[5] FLT was ranged from 48 to 55 s, AR9 formulation showed maximum FLT and AR7 showed the minimum value as shown in Table 5. TFT of all prepared formulations was found to be greater than 8 h.

An *in vitro* method reported by Jang et al. [7] were used to check the buffering and neutralizing capacity. pH values after 4 and 20 min were recorded as mentioned in Table 5. The formulations containing the maximum amount of sodium citrate and citric acid possessed higher buffering between pH 2.5–4.5 and neutralizing capacity. The pH after 4 and 20 min checks an un-physiologically high pH and neutralizing capacity between 2.5 and 4.5 is sign for the efficacy in the physiological environment.

The drug release from alginate rafts forming formulations AR1–AR15 were investigated. The concentration of sodium alginate was ranged from 10 to 40% have an effect on release of drug from raft. When the amount of sodium alginate was increased the release of PSS from raft was decreased. PSS is freely water soluble, a retardant HPMC K100M was added to sustain the release pattern of drug from raft. HPMC K100M form a gel barrier around raft that allows the drug to be released by diffusion process. HPMC K100M was used the concentration ranges of 6–14%. As predictable, on increasing the concentration of HPMC K100M, the thickness of gel barrier was increased that delayed the release of PSS from raft. He et al. studied the effect of effect of HPMC K100 on release profile of metformin.[4] Sodium bicarbonate 20–40% a gas generating substance also have effect on drug release from raft.[17] Sodium bicarbonate generate carbon dioxide after reacting with acidic dissolution medium and resulted in the form of gel like raft system at the surface of the medium. The carbon dioxide is entrapped in the gel cause obstruction of diffusion pathway of drug release from raft. This effect was more observed at low polymer concentrations in the formulation (AR1, AR13). When the polymer concentrations were increased in the formulation (AR2, AR5, AR9) the effect of sodium bicarbonate on drug release from alginate raft was decreased. Jiménez-Martínez et al. studied the effect of sodium bicarbonate on release profile of captopril from floating matrix tablets.[18] The drug release percentages after 2, 4 and 8 h were mentioned in Table 2. The optimized formulation AR9 showed optimum drug release i.e. 98%.

In kinetic release models *R* ^2^ values of zero order release were ranged from 0.711 to 0.981 while in first order release it was 0.957–0.990 and which observed the concentration dependent release of PSS. In korsmeyer-peppas model value of n was found to be 0.57 which was greater than 0.45 showing a non-fickian drug release mechanism in prepared formulations. Jose et al. studied the release pattern of insulin from tablets of crosslinked chitosan microspheres by using korsmeyer-peppas.[19]

FTIR spectra of PSS showed its features peaks at 1587.83, 1301.87, 1104.89 cm^−1^ due to C=C stretching, C–N stretching and C–F stretching, respectively.[20] Sodium alginate showed peaks at 1583.96, 1406.68 cm^−1^ provide information about water and carboxylic group of alginate and 1022.38 cm^−1^ due to –OH bending vibration.[21] HPMC K100M showed two peaks at 1054.87 and 3400.03 cm^−1^ due to C–O and O–H stretching vibrations respectively.[22]). Raft of AR9 formulation showed peaks at 3223.09, 1688.17, 1586.67^−1^, 1302.64^−1^, 1162.01^−1^,and 1027.74 cm^−1^ due to –OH stretching, ester carbonyl group (C=O) stretching, existence of water and carboxylic group in raft, C–N stretching, C–F stretching and –OH bending vibration. The FTIR spectra of drug, polymers and raft of AR9 formulation showed no notable interaction between them. Spectra of raft of AR9 formulation showed the presence of sodium alginate, HPMC K100M and drug were effectively entrapped in the pectin raft.

DSC thermograms of PSS, sodium alginate, HPMC K100M, tablet of optimized formulation AR9 and raft of AR9 formulation are shown in Figure 4. Thermogram of PSS showed an endothermic peaks at 250 °C which was the indication of melting point of PSS.[23] Thermograms of sodium alginate, HPMC K100M, tablet of optimized formulation AR9 and raft of AR9 optimized formulation showed no peaks indicating that PSS was dispersed in the tablet and raft effectively.

XRD diffractograms showed characteristics diffraction lines of PSS at 2*θ* of 6°and 22° due to its crystalline nature are shown in Figure 5.[23] Sodium alginate showed well defined peaks at 3° (2*θ*) related to its crystallinity due to strong intermolecular hydrogen bonding [24] and HPMC K100M at 3°, 9° and 18° (2*θ*).[25] The diffractograms of tablet of optimized formulation AR9 and raft of AR9 optimized formulation showed many characteristics peaks at 15°, 17°, 19°, 27°, 32° and 40° (2*θ*) but disappearance of the peaks of PSS, sodium alginate and HPMC K100M were observed. This indicated that the crystalline nature of PSS was decreased after tablet preparation and raft formation of AR9 optimized formulation.

## 4. Conclusion

Raft forming tablets were successfully developed using sodium alginate as raft forming polymers, HPMCK100M for sustained effect, sodium bicarbonate and citric acid as gas generating agents and neutralizing agent calcium carbonate. This novel oral dosage form rapidly disintegrate and formed floating raft on the surface of SGF, preventing reflux disorders associated with peptic ulcer and release the PSS up to 8 h. The raft floats on the surface of SGF for up to 24 h with 1 min of FLT. *In vitro* modified balance method for measurement of raft strength was developed successfully. Optimized formulation AR9 showed good strength, thickness and resilience of raft.

## Disclosure statement

No potential conflict of interest was reported by the authors.

## References

[1] Diós P, Nagy S, Pál S, et al Preformulation studies and optimization of sodium alginate based floating drug delivery system for eradication of *Helicobacter pylori* . Eur. J. Pharm. Biopharm. 2015;96:196–206. 10.1016/j.ejpb.2015.07.020 26247118

[2] Mukhopadhyay P, Sarkar K, Soam S, et al Formulation of pH-responsive carboxymethyl chitosan and alginate beads for the oral delivery of insulin. J. Appl. Polym. Sci. 2013;129:835–845. 10.1002/app.38814

[3] Ghayempour S, Mortazavi SM Preparation and investigation of sodium alginate nanocapsules by different microemulsification devices. J. Appl. Polym. Sci. 2015;132:41904–41911.

[4] He W, Li Y, Zhang R, et al Gastro-floating bilayer tablets for the sustained release of metformin and immediate release of pioglitazone: Preparation and in vitro/in vivo evaluation. Int. J. Pharm. 2014;476:223–231. 10.1016/j.ijpharm.2014.09.056 25283698

[5] Hampson F, Farndale A, Strugala V, et al Alginate rafts and their characterisation. Int. J. Pharm. 2005;294:137–147. 10.1016/j.ijpharm.2005.01.036 15814238

[6] Hampson FC, Jolliffe IG, Bakhtyari A, et al Alginate–antacid combinations: raft formation and gastric retention studies. Drug Dev. Ind. Pharm. 2010;36:614–623. 10.3109/03639040903388290 19925256

[7] Jang SW, Lee JW, Ryu DS, et al Design of pH-responsive alginate raft formulation of risedronate for reduced esophageal irritation. Int. J. Biol. Macromol. 2014;70:174–178. 10.1016/j.ijbiomac.2014.06.048 24995633

[8] Kerdsakundee N, Mahattanadul S, Wiwattanapatapee R Development and evaluation of gastroretentive raft forming systems incorporating curcumin-Eudragit® EPO solid dispersions for gastric ulcer treatment. Eur. J. Pharm. Biopharm. 2015;94:513–520. 10.1016/j.ejpb.2015.06.024 26143367

[9] Rapolu K, Sanka K, Vemula PK, et al Optimization and characterization of gastroretentive floating drug delivery system using Box-Behnken design. Drug Dev. Ind. Pharm. 2013;39:1928–1935. 10.3109/03639045.2012.699068 22762132

[10] Sharma D, Maheshwari D, Philip G, et al Formulation and optimization of polymeric nanoparticles for intranasal delivery of lorazepam using Box-Behnken design: in vitro and in vivo evaluation. BioMed Res. Int. 2014;2014:1–14. 10.1155/2014/156010 PMC4122152 25126544

[11] Mandal U, Pal TK Formulation and in vitro studies of a fixed-dose combination of a bilayer matrix tablet containing metformin HCl as sustained release and glipizide as immediate release. Drug Dev. Ind. Pharm. 2008;34:305–313. 10.1080/03639040701657487 18363146

[12] Elliott BM, Steckbeck KE, Murray LR, et al Rheological investigation of the shear strength, durability, and recovery of alginate rafts formed by antacid medication in varying pH environments. Int. J. Pharm. 2013;457:118–123. 10.1016/j.ijpharm.2013.09.034 24095816

[13] Prajapati ST, Mehta AP, Modhia IP, et al Formulation and optimisation of raft-forming chewable tablets containing H2 antagonist. Int. J. Pharm. Invest. 2012;2:176–182. 10.4103/2230-973X.106988 PMC3618633 23580933

[14] Prajapati VD, Jani GK, Khutliwala TA, et al Raft forming system – an upcoming approach of gastroretentive drug delivery system. J. Controlled Release. 2013;168:151–165. 10.1016/j.jconrel.2013.02.028 23500062

[15] Bose A, Wong TW, Singh N Formulation development and optimization of sustained release matrix tablet of Itopride HCl by response surface methodology and its evaluation of release kinetics. Saudi Pharm. J. 2013;21:201–213. 10.1016/j.jsps.2012.03.006 23960836 PMC3744972

[16] Dettmar P, Hampson F, Farndale A, et al Alginate rafts and their characterization. Int. J. Pharm. 2005;294:137–147. 15814238 10.1016/j.ijpharm.2005.01.036

[17] Bhandari PN, Jones DD, Hanna MA Characterization of sodium starch glycolate prepared using reactive extrusion and its comparisons with a commercial brand VIVASTAR® P. Ind. Crops Prod. 2013;41:324–330. 10.1016/j.indcrop.2012.04.050

[18] Jiménez-Martínez I, Quirino-Barreda T, Villafuerte-Robles L Sustained delivery of captopril from floating matrix tablets. Int. J. Pharm. 2008;362:37–43. 10.1016/j.ijpharm.2008.05.040 18588962

[19] Jose S, Fangueiro J, Smitha J, et al Predictive modeling of insulin release profile from cross-linked chitosan microspheres. Eur. J. Med. Chem. 2013;60:249–253. 10.1016/j.ejmech.2012.12.011 23313633

[20] Reddy GM, Bhaskar BV, Reddy PP, et al Structural identification and characterization of potential impurities of pantoprazole sodium. J. Pharm. Biomed. Anal. 2007;45:201–210. 10.1016/j.jpba.2007.05.032 17629653

[21] Borba PAA, Pinotti M, de Campos CEM, et al Sodium alginate as a potential carrier in solid dispersion formulations to enhance dissolution rate and apparent water solubility of BCS II drugs. Carbohydr. Polym. 2016;137:350–359. 10.1016/j.carbpol.2015.10.070 26686139

[22] Ding C, Zhang M, Li G Preparation and characterization of collagen/hydroxypropyl methylcellulose (HPMC) blend film. Carbohydr. Polym. 2015;119:194–201. 10.1016/j.carbpol.2014.11.057 25563960

[23] Zupančič V, Ograjšek N, Kotar-Jordan B, et al Physical characterization of pantoprazole sodium hydrates. Int. J. Pharm. 2005;291:59–68. 10.1016/j.ijpharm.2004.07.043 15707732

[24] Seeli DS, Dhivya S, Selvamurugan N, et al Guar gum succinate-sodium alginate beads as a pH-sensitive carrier for colon-specific drug delivery. Int. J. Biol. Macromol. 2016;91:45–50. 10.1016/j.ijbiomac.2016.05.057 27212216

[25] Meneguin AB, Cury BSF, Evangelista RC Films from resistant starch-pectin dispersions intended for colonic drug delivery. Carbohydr. Polym. 2014;99:140–149. 10.1016/j.carbpol.2013.07.077 24274490

